# Colorectal Cancer Screening With High Risk-Factor Questionnaire and Fecal Immunochemical Tests Among 5, 947, 986 Asymptomatic Population: A Population-Based Study

**DOI:** 10.3389/fonc.2022.893183

**Published:** 2022-05-30

**Authors:** Mingqing Zhang, Lizhong Zhao, Yongdan Zhang, Haoren Jing, Lianbo Wei, Zhixuan Li, Haixiang Zhang, Yong Zhang, Siwei Zhu, Shiwu Zhang, Xipeng Zhang

**Affiliations:** ^1^ Nankai University School of Medicine, Nankai University, Tianjin, China; ^2^ Department of Colorectal Surgery, Tianjin Union Medical Center, Tianjin, China; ^3^ Colorectal Cancer Screening Office, Tianjin Institute of Coloproctology, Tianjin, China; ^4^ The Institute of Translational Medicine, Tianjin Union Medical Center of Nankai University, Tianjin, China; ^5^ Center for Applied Mathematics, Tianjin University, Tianjin, China; ^6^ Department of Pathology, Tianjin Union Medical Center, Tianjin, China

**Keywords:** high-risk factor questionnaire, fecal immunochemical test, colorectal cancer screening, colonoscopy, asymptomatic population

## Abstract

**Background:**

The recent uptrend in colorectal cancer (CRC) incidence in China is causing an increasingly overwhelming social burden. And its occurrence can be effectively reduced by sensitizing CRC screening for early diagnosis and treatment. However, a large number of people in China do not undergo screening due to multiple factors. To address this issue, since 2012, a CRC screening program has been initiated in Tianjin.

**Methods:**

Residents aged 40-74 years were eligible for CRC screening. The first was to complete the high-risk factor questionnaire (HRFQ) and undergo fecal immunochemical test (FIT). Then those with a positive result in any of the two screening methods were recommended for a free colonoscopy.

**Results:**

The detection rate of intestinal diseases increased with age, had a male predominance, and was higher in residents from central urban areas and those with primary school above education level. The sensitivity of predicting CRC after colonoscopy in the high-risk group was 76.02%; the specificity was 25.33%.A significant decrease in the detection rate of intestinal disease, CRC and advanced adenoma was observed from positive FIT, the high-risk group and positive HRFQ, 47.13%, 44.79%, 42.30%; 3.15%, 2.44%, 1.76%; 7.72%, 6.42%, 5.08%, in that order, while no inter-group difference was found for the detection of polyps. In addition, the different combinations of HRFQ and FIT can enroll more high-risk population than FIT or (and) HRFQ only, and thus detect more intestinal diseases (include CRC/AA/Polyp).

**Conclusion:**

The superimposition of different screening method for HRFQ and FIT is an effective strategy for the detection of CRC, AA, and Polyp, compared to HRFQ or FIT alone. However, further improvements in screening and interventions are needed to promote colonoscopy compliance.

## 1 Introduction

Colorectal cancer (CRC) is the third most common cancer and the second leading cause of cancer death worldwide (1). However, CRC can be preventable *via* screening due to its long development time from precancerous (i.e., polyps and advanced adenomas [AA]) to cancerous lesion and may have a relatively good prognosis if diagnosed and treated early (2, 3). Screening remains the most powerful public health tool for reducing CRC incidence and mortality (4). Studies have shown that the fecal immunochemical test (FIT) could reduce CRC incidence and mortality rates by 10% and 22%-62% (5–7), while the reduction in incidence and mortality rates by colonoscopy could be substantially higher at 31%-69% and 29%-67%, respectively (8–10). Despite the benefits of screening, its effectiveness is influenced by many factors, among which participants’ engagement and compliance are the two most important contributors. Currently, FIT is the most common screening test for CRC worldwide (11, 12) but its accuracy could be limited due to fecal hemoglobin degradation, intermittent bleeding and non-bleeding lesions.

In China, CRC screening has been implemented since the 1970s. Based on the CRC general census that was performed twice in Jiashan County and Haining County of the Zhejiang Province, the high-risk factors questionnaire (HRFQ) was developed for CRC screening in China (13). HRFQ can be used in any asymptomatic population based on epidemiological risk factors, which tends to increase the screening population and covers a shortage in FITs. Previous studies have demonstrated that the combination of questionnaire and fecal occult blood test/fecal immunochemical test (FOBT/FIT) could be the optimal screening method for China and an effective strategy among economically and medically underserved populations (14–18). Thus, this combined screening method has been implemented in many cities, such as Guangzhou, Shanghai, and Hangzhou, across China and has achieved impactful results.

Since 2012, CRC screening in community allied third-grade class-A hospital has been initiated in the Tianjin city of China and has already completed three rounds of screening; representing one of the largest screening programs in China. In this study, we present the results of this CRC screening program based on the findings from HRFQ, FIT and colonoscopy that were performed from 2012 to 2020 in Tianjin city, evaluated the implementation of the screening program and determined its impact on CRC diagnosis and possible prevention.

## 2 Methods

### 2.1 Study Population

The screening protocol was developed by Tianjin CRC Screening Office and conducted in Tianjin primary care units and medical institutions performing colonoscopy. The CRC screening was free of charge for permanent residents aged 40-74 years old and was performed in a 3-year cycle between 2012 and 2020. with the first cycle from 2012 to 2014, in which the main screening population is 70-74 years old in 2012, 50-60 years old in 2013, and 40-50 years old in 2014. And so on, 2015 -2017 for the second cycle of screening work, 2018-2020 for the third cycle of screening work. All participating primary care units were required to sensitize eligible people within their respective jurisdictions to first complete the questionnaire survey then undergo FIT, in an orderly manner. Participants that were identified as high-risk based on the HRFQ and FIT results were suggested to undergo colonoscopy at designated hospitals through advice notes issued by the screening physicians. At the same time, the primary care units were responsible for the follow-up of these high-risk people. All the screening-related testing was conducted in CRC screening units designated by Tianjin Health Commission. HRFQ, FIT and colonoscopy were free. After eliminating erroneous cases, defined as missing values and outliers, the data from the 2012-2020 CRC screening were collected and used for the final analysis of this study. The demographic information of the participants and their FIT and colonoscopy results were obtained from the Tianjin CRC Screening Database.

### 2.2 Screening Protocol

The screening strategy during the 9-year study was performed in two steps. First, after providing an informed consent, all participants were asked to complete the HRFQ followed by FIT. The positive HRFQ was defined as participants meeting any of the following conditions: a) a history of CRC in a first-degree relative; b) history of cancer or intestinal polyps; c) history of two or more chronic constipation, chronic diarrhea, mucous bloody stools, adverse life events (e.g., divorce, death of a close relative, etc.), chronic appendicitis or appendectomy and chronic cholecystitis or gallstones. High risk groups that were defined as positive-HRFQ or positive FIT. Second, those who had positive HRFQ or (and) FIT results were advised to undergo subsequent colonoscopy, whereby biopsy and/or polyp removal was performed when needed.

### 2.3 FIT

Fecal occult blood was detected using the immunogold method and the reagents were provided by Abbott Biotechnology Co., Ltd. Based on a pre-arranged date and without any restriction on diet, each participant was asked to provide 10-50 mg of stool sample which was sent to a corresponding screening hospital laboratory on the day of collection and were analyzed within 8 hours of collection. Following the manufacturer’s instructions (Abbott), the results were qualitatively reported by a central laboratory, that is, either as being positive or negative. Lastly, 4% of the stool samples were randomly selected for quality control of the FIT results.

### 2.4 Colonoscopy Screening

Participants who had positive HRFQ or FIT results were interviewed by the screening physicians and were recommended to undergo colonoscopy. Their basic information, such as age, gender, occupation, region, education level, and medical history data were also recorded.

### 2.5 Ethics

The colorectal cancer screening protocol was approved by the local ethical committee in the Health Bureau of Tianjin City. And all investigations and methods used were in accordance with the Declaration of Helsinki.

### 2.6 Data Collection and Results Measurement

Endoscopic and histopathological data from colonoscopy were recorded in a dedicated database. CRC was defined as adenocarcinoma of the colon or rectum. AA was defined as adenomas of diameter ≥1cm, villous adenomas with at least 25% of villous components, or adenomas with severe dysplasia. Although the quality of colonoscopy could not be truly standardized due to possible subjective differences between individual endoscopists, however, all the endoscopists involved in the screening program had extensive clinical experience in colonoscopy. The presence of polyps, AAs or CRC by colonoscopy was classified as a positive test result for intestinal disease, while those without any significant abnormalities on colonoscopy were classified as negative for intestinal disease.

### 2.7 Evaluation Indicators

Positive rate (%): The proportion of participants who had positive test results. HRFQ positive rate = number of positive HRFQs/number of the overall HRFQs. FIT positive rate = number of positive FITs/number of the overall FITs samples.

Compliance rate of subsequent examinations (%): Post-HRFQ FIT compliance rate = number of FITs/number of HRFQs. Positive FIT/HRFQ/high-risk group subsequent colonoscopy compliance rate = number of colonoscopies performed/number of Positive FIT/HRFQ/high-risk participants.

### 2.8 Statistical Analysis

Enumeration data are described by the number of cases or constituent ratio. Numerical differences between groups were assessed using the chi-square test. The threshold for significance was *P*<0.05. Age was divided into 4 groups, which had separate thresholds, for comparisons between age groups, the significance threshold was *P* < 0.0083. All statistical analyses were conducted using the SPSS software (version 24.0; SPSS Inc., Chicago, IL, USA).

## 3 Results

### 3.1 Characteristics of the Study Population

**Figure 1:**
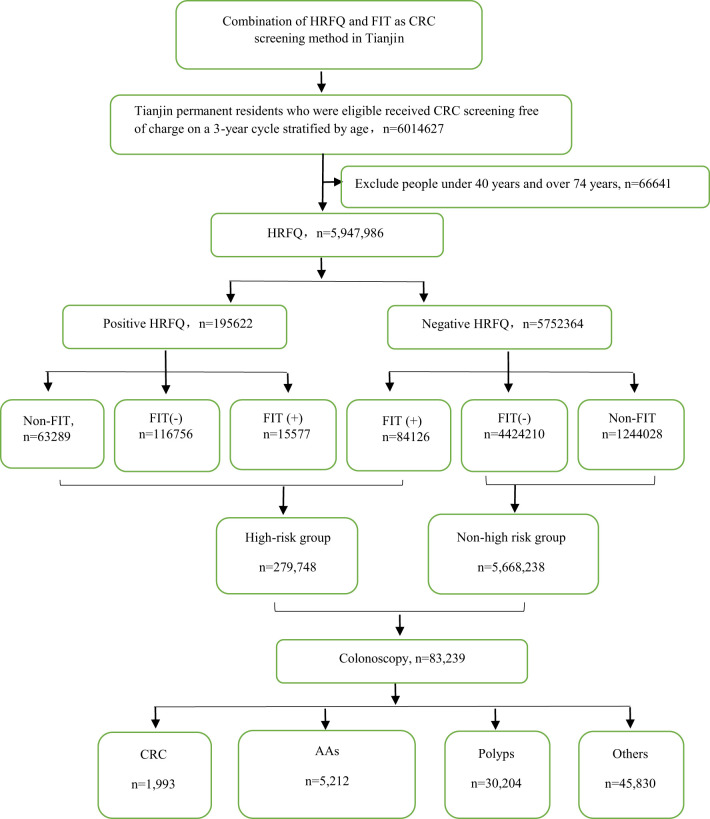
Research schematic of colorectal cancer screening for Tianjin permanent residents.

From 2012 to 2020, a total of 5,947,986 participants completed the HRFQ and 4,640,669 participants underwent FIT in Tianjin. The number of participants who were identified as high-risk in the first screening stage was 279,748, of whom 195,622 (3.29%) had positive HRFQ and 99,703 (2.15%) had positive FIT results. Further analyses of the high-risk population revealed that 15577 (5.57%) had both positive HRFQ and FIT results (**Figure 1**). In the second stage of screening, 83,239 participants underwent colonoscopy following the screening physicians’ recommendations. The proportion of HRFQ, FIT, and subsequent colonoscopy was significantly higher in women and people aged 60-69 and 50-59 years old (**Table 1**).

**Table 1:** Characteristics of the study population of the Tianjin Colorectal Cancer Screening Project (2012-2020).

	Positive HRFQ (n)	Negative HRFQ (n)	Total(n)	Total (%)	Positive FIT(n)	Negative FIT(n)	Total(n)	Total(%)	HRFQ(+)+FIT(+)(n)	Total(%)	Positive colonoscopy (n)	Negative colonoscopy (n)	Total(n)	Total (%)
Gender														
Male	80221	2727416	2807637	47.20	47405	2129025	2176430	46.90	7041	45.20	21083	17987	39070	46.94
Female	115401	3024948	3140349	52.80	52298	2411941	2464239	53.10	8536	54.80	16326	27843	44169	53.06
Age group														
40-49	23037	1360711	1383748	23.26	13722	1080023	1093745	23.57	2312	14.84	2925	6289	9214	11.07
50-59	56805	1802620	1859425	31.26	29197	1489216	1518413	32.72	5013	32.18	10959	15584	26543	31.89
60-69	90259	2027387	2117756	35.60	43926	1547971	1591897	34.30	6502	41.74	19415	20663	40078	48.15
70-74	25521	561646	587167	9.87	12858	423756	436614	9.41	1750	11.23	4110	3294	7404	8.89
Year														
2012-2014	95016	2009668	2104684	35.38	37415	1558701	1596116	34.39	6175	39.64	17128	26534	43662	52.45
2015-2017	49193	2047836	2097029	35.26	29273	1678674	1707947	36.80	4457	28.61	10664	10552	21216	25.49
2018-2020	51413	1694860	1746273	29.36	33015	1303591	1336606	28.80	4945	31.75	9617	8744	18361	22.06
2012-2020	195622	5752364	5947986	100.0%	99703	4540966	4640669	100.0%	15577	100.0%	37409	45830	83239	100.0%

### 3.2 Evaluation of the CRC Screening Program Results

#### 3.2.1 HRFQ Results

**Figure 2:**
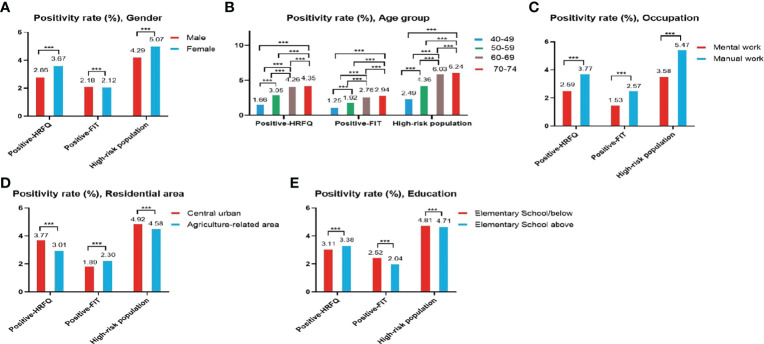
Evaluation of the CRC screening program results by gender **(A)**, age group **(B)**, occupation **(C)**, residential area **(D)**, education **(E)**. ***There are significant differences in detection rates between the different screening methods.

The proportion of females who tested positive on the HRFQ was higher than in males (3.67% vs. 2.86%, respectively) (**Table 2** and **Figure 2A**). The positive rate in the 70-74, 60-69, 50-59, and 40-49 age groups was 4.35%, 4.26%, 3.05%, and 1.66%, respectively (**Table 2** and **Figure 2B**). Further, the positive rate was higher in manual workers (3.77%) than those in mental work (2.59%) and higher in participants from central urban areas (3.77%) than those from agriculture-related areas (3.01%) (**Table 2** and **Figures 2C, D**). In regard to education level, the positive rate was higher in participants who attended primary school above (3.38%) than those who had lower education (3.11%).(**Table 2** and **Figure 2E**).

**Table 2:** Comparison of positive HRFQ for different CRC screening population subgroups (2012-2020), (n, %).

		HRFQ		Total	chi-square	*P* value
		Positive	Negative			
Gender	Male	80221 (2.86)	2727416 (97.14)	2807637	3114.891	<0.001
	Female	115401 (3.67)	3024948 (96.33)	3140349		
Age group	40-49	23037 (1.66)	1360711 (98.34)	1383748	20166.759	<0.001
	50-59	56805 (3.05)	1802620 (96.95)	1859425		
	60-69	90259 (4.26)	2027387 (95.74)	2117646		
	70-74	25521 (4.35)	561646 (95.65)	587167		
Education	Elementary School/below	48278 (3.11)	1502036 (96.89)	1550314	255.122	<0.001
	Elementary school above	146248 (3.38)	4178569 (96.62)	4324817		
Occupation	mental work	61171 (2.59)	2296362 (97.41)	2357533	6111.076	<0.001
	manual work	133946 (3.77)	3421265 (96.23)	3555211		
Residential area	central urban	82628 (3.77)	2110411 (96.23)	2193039	2480.302	<0.001
	agriculture-related areas	112977 (3.01)	3637366 (96.99%)	3750343		

HRFQ missing values are 72855 for education; 39622 for occupation; and 4604 for residential area.

#### 3.2.2 FIT Results

The positive rate of FIT in males (2.18%) was higher than that in females (2.12%) (**Table 3** and **Figure 2A**). The positive rate of FIT in the 70-74, 60-69, 50-59, and 40-49 age groups was 2.94%, 2.76%, 1.92%, and 1.25%, respectively (**Table 3** and **Figure 2B**). Further, the positive rate was higher in manual workers (2.57%) than those in mental work (1.53%) (**Table 3** and **Figure 2C**), and was higher in participants from agriculture-related areas (2.30%) than those from central urban areas (1.89%) (**Table 3** and **Figure 2D**). In regard to education level, the positive rate of FIT was lower in participants who attended primary school above (2.04%) than those who had lower education (2.52%) (**Table 3** and **Figure 2E**).

**Table 3:** Comparison of positive FIT for different CRC screening population subgroups (2012-2020), (n, %).

		FIT		Total	chi-square	*P* value
		Positive	Negative			
Gender	Male	47405 (2.18)	2129025 (97.82)	2176430	17.135	<0.001
	Female	52298 (2.12)	2411941 (97.88)	2464239		
Age group	40-49	13722 (1.25)	1080023 (98.75)	1093745	8667.812	<0.001
	50-59	29197 (1.92)	1489216 (98.08)	1518413		
	60-69	43926 (2.76)	1547971 (97.24)	1591897		
	70-74	12858 (2.94)	423756 (97.06)	436614		
Education	Elementary School/below	30033 (2.52)	1162370 (97.48)	1192403	952.495	<0.001
	Elementary school above	69224 (2.04)	3323249 (98.96)	3392473		
Occupation	mental work	28014 (1.53)	1801322 (98.47)	1829336	5613.144	<0.001
	manual work	71490 (2.57)	2713374 (97.43)	2784864		
Residential area	central urban	31974 (1.89)	1664176 (98.11)	1696150	898.855	<0.001
	agriculture-related areas	67721 (2.30)	2870896 (97.70)	2938617		

Missing values for FIT are 55,793 for education; 26,469 for occupation; and 5902 for residential area.

#### 3.2.3 High-Risk Population

The high-risk population rate of females (5.07%) than males (4.29%) (**Table 4-1** and **Figure 2A**). The proportion of high-risk participants in the70-74, 60-69, 50-59, and 40-49 age groups was 6.24%, 6.03%, 4.36%, and 2.49%, respectively (**Table 4-1** and **Figure 2B**). The proportion of high-risk participants was higher among manual workers (5.47%) than among those doing mental work (3.58%) (**Table 4-1** and **Figure 2C**) and was also higher in central urban areas (4.92%) than in agriculture-related areas (4.58%) (**Table 4-1** and **Figure 2D**).The proportion of high-risk participants was higher in those who had an educational level below primary school (4.81%) than those who attended primary school above (4.71%) (**Table 4-1** and **Figure 2E**). The demographic characteristics of the high-risk population and the comparison of colonoscopies and non-colonoscopies among the high-risk population are detailed in **Table 4-2**.

**Table 4-1:** Comparison of high-risk population for different CRC screening populations (2012-2020), (n, %).

		CRC screening populations		Total	chi-square	P value
		High-risk population	Non high-risk population			
Gender	Male	120585 (4.29)	2687052 (95.71)	2807637	1978.404	<0.001
	Female	159163 (5.07)	2981186 (94.93)	3140349		
Age group	40-49	34447 (2.49)	1349301 (97.51)	1383748	27030.372	<0.001
	50-59	80989 (4.36)	1778436 (95.64)	1859425		
	60-69	127684 (6.03)	1989962 (93.97)	2117646		
	70-74	36629 (6.24)	550538 (93.76)	587167		
Education	Elementary School/below	74523 (4.81)	1475791 (95.19)	1550314	23.022	<0.001
	Elementary school above	203766 (4.71)	4121051 (95.29)	4324817		
Occupation	mental work	84493 (3.58)	2273040 (96.42)	2357533	11250.570	<0.001
	manual work	194584 (5.47)	3360627 (94.53)	3555211		
Residential area	central urban	107962 (4.92)	2085077 (95.08)	2193039	363.145	<0.001
	agriculture-related areas	171761 (4.58)	3578582 (95.42)	3750343		

**Table 4-2:** Comparison of colonoscopy and non colonoscopy in high-risk population (2012-2020), (n, %).

		High-risk population		Total	chi-square	P value
		colonoscopy	Non colonoscopy			
Gender	Male	29172 (46.92)	91413 (42.02)	120585 (43.10)	473.363	<0.001
	Female	33008 (53.08)	126155 (57.98)	159163 (56.90)		
Age group	40-49	6701 (10.78)	27746 (12.76)	34447 (12.31)	1524.683	<0.001
	50-59	19867 (31.95)	61122 (28.09)	80989 (28.95)		
	60-69	29993 (48.23)	97691 (44.90)	127684 (45.64)		
	70-74	5619 (9.04)	31010 (14.25)	36629 (13.10)		
Education	Elementary School/below	14275 (23.09)	60248 (27.83)	74523 (26.78)	551.097	<0.001
	Elementary school above	47544 (76.91)	156222 (72.17)	203766 (73.22)		
Occupation	mental work	18205 (29.39)	66288 (30.53)	84493 (30.28)	29.377	<0.001
	manual work	43731 (70.61)	150853 (69.47)	194584 (69.72)		
Residential area	central urban	25433 (40.90)	82529 (37.94)	107962 (38.60)	178.833	<0.001
	agriculture-related areas	36753 (59.10)	135008 (62.06)	171761 (61.40)		

Missing values for occupation are 671; missing values for residential area are 25; missing values for education are 1459.

### 3.3 Compliance Evaluation of the Tianjin CRC Screening Program

#### 3.3.1 Compliance Results of FIT After HRFQ

**Figure 3:**
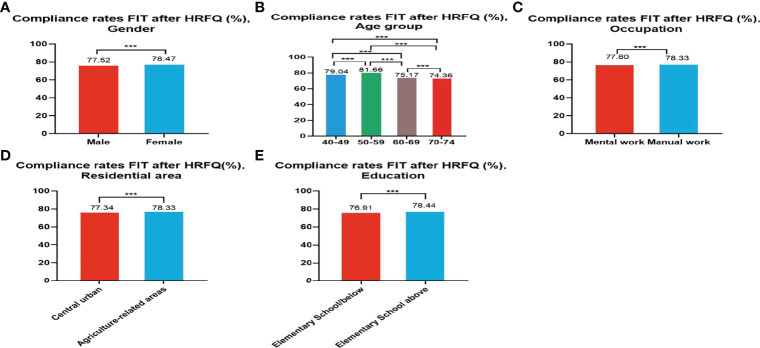
Compliance results by gender **(A)**, age group **(B)**, occupation **(C)**, residential area **(D)**, education **(E)** of FIT after HRFQ. ***There are significant differences in detection rates between the different screening methods.

Of the 5,947,986 participants who completed the HRFQ of the screening program from 2012 to 2020, 4,640,669 participants underwent FIT; demonstrating a completion rate of 78.02%. The compliance rate for FIT in females (78.47%) was higher than in males (77.52%) (**Table 5** and **Figure 3A**). In descending order, the compliance rate for FIT in the 50-59, 40-49, 60-69, and 70-74 age groups was 81.66%, 79.04%, 75.17%, and 76.91%, respectively(**Table 5** and **Figure 3B**). Further, the compliance rate to undergo FIT was higher in manual workers (78.33%) than those doing mental work (77.60%) (**Table 5** and **Figure 3C**), higher in participants from agriculture-related areas (78.36%) than from central urban areas (77.34%) (**Table 5** and **Figure 3D**), and higher in those who had primary school above education level (76.91%) than those with lower education (2.04%) (**Table 5** and **Figure 3E**).

**Table 5:** Analysis of compliance results of FIT after HRFQ, (n,%).

Year	HRFQ	FIT	①^*^ (%)	Gender		Age group				Occupation		Residential area		Education	
				Male	Female	40-49	50-59	60-69	70-74	mental work	manual work	②^*^	③^*^	④^*^	⑤^*^
2012-2014	2104684	1596116	75.84	752179 (75.45)	843943 (76.19)	449596 (84.35)	551702 (86.79)	473767 (64.15)	121051 (61.32)	960104 (75.67)	629265 (76.11)	603734 (72.49)	990292 (78.01)	468389 (73.14)	1123997 (76.98)
2015-2017	2097029	1707947	81.45	807963 (80.99)	899984 (81.86)	441432 (76.19)	521363 (82.75)	605681^#^ (83.99)	139471^#^ (83.76)	501662 (82.81)	1195636 (80.88)	647820 (83.73)	1057941 (79.97)	415068 (80.91)	1272785 (81.61)
2018-2020	1746273	1336606	76.54	616294 (75.80)	720312 (77.19)	202717 (74.70)	445348 (75.01)	512449 (77.88)	176092 (78.88)	367570 (76.11)	959963 (77.06)	444596 (75.81)	890384 (76.89)	308946 (77.83)	995691 (76.29)
2012-2020	5947986	4640669	78.02	2176436 (77.52)	2464239 (78.47)	1093745 (79.04)	1518413 (81.66)	1591897 (75.17)	436614 (74.36)	1829336 (77.60)	2784864 (78.33)	1696150 (77.34)	2938617 (78.36)	1192403 (76.91)	3392473 (78.44)

missing values for occupation are 26469; missing values for residential area are 5902; missing values for education are 55793;

①*:compliance results of FIT after HRFQ; ②^*^:central urban; ③^*^:agriculture-related areas; ④^*^:Elementary School/below; ⑤^*^:Elementary school above;

^#^No significant differences were observed between groups; And statistically significant differences were detected between the other groups.

#### 3.3.2 Compliance Results of Subsequent Colonoscopy in the High-Risk Population

**Figure 4:**
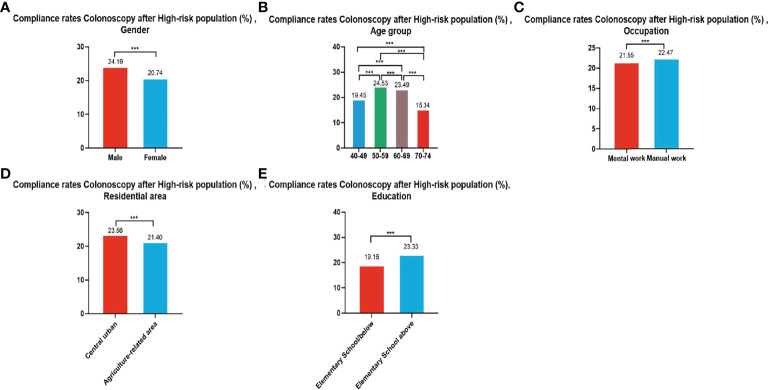
Compliance results by gender **(A)**, age group **(B)**, occupation **(C)**, residential area **(D)**, education **(E)** of subsequent colonoscopy in the high-risk population. ***There are significant differences in detection rates between the different screening methods.

In all, 279,748 participants were identified as high-risk in the first stage of screening from 2012 to 2020, of whom 62,180 underwent colonoscopies; demonstrating a compliance rate of 22.23%. The compliance rate for colonoscopy was higher in males (24.19%) than in females (20.74%) (**Table 6** and **Figure 4A**). In descending order, the compliance rate in the 50-59, 60-69, 40-49, and 70-74 age groups was 24.53%, 23.49%, 19.45%), and 15.34%, respectively (**Table 6** and **Figure 4B**). Further, the compliance rate in participants who had primary school above education level (23.33%) was higher than those with lower education (19.16%) (**Table 6**, **Figure 4E**), higher in manual workers (22.47%) than in those doing mental work (21.55%) (**Table 6** and **Figure 4C**), and was also higher in central urban areas (23.56%) than in agriculture-related areas (21.40%) (**Table 6** and **Figure 4D**).

**Table 6:** Analysis of compliance results of subsequent colonoscopy in the high-risk population, (n,%).

Year	High-risk population	Colonoscopy	①^*^ (%)	Gender		Age group				Occupation		Residential area		Education	
				Male	Female	40-49	50-59	60-69	70-74	mental work	manual work	②^*^	③^*^	④^*^	⑤^*^
2012-2014	126256	32938	26.09	14956 (27.96)	17982 (24.71)	3520 (22.24)	10343^#^ (28.35)	15911^#^ (27.63)	3164 (19.33)	10472 (24.62)	22338 (26.73)	12843# (26.38)	20104# (25.92)	8599 (22.38)	24205 (27.61)
2015-2017	74009	15331	20.72	7580 (23.06)	7751 (18.84)	2413 (21.44)	5005 (24.11)	6805 (19.97)	1108 (13.98)	3802 (19.25)	11519 (21.24)	6185 (21.97)	9146 (19.95)	3173 (16.52)	12148 (22.17)
2018-2020	79483	13911	17.50	6636 (19.39)	7275 (16.08)	768^#^ (10.43)	4519 (19.03)	7277 (20.20)	1347^#^ (10.92)	3931# (17.70)	9874# (17.40)	6405 (20.58)	7503 (15.52)	2503 (14.82)	11191 (18.26)
2012-2020	279748	62180	22.23	29172 (24.19)	33008 (20.74)	6701 (19.45)	19867 (24.53)	29993 (23.49)	5619 (15.34)	18205 (21.55)	43731 (22.47)	25433 (23.56)	36753 (21.40)	14275 (19.16)	47544 (23.33)

missing values for occupation are 244; missing values for residential area are 4; missing values for education are 361;

①^*^: compliance results of subsequent colonoscopy in the high-risk population; ②^*^:central urban; ③^*^:agriculture-related areas; ④^*^:Elementary School/below; ⑤^*^:Elementary school above;

^#^No significant differences were observed between groups; And statistically significant differences were detected between the other groups.

#### 3.3.3 Compliance Results of Subsequent Colonoscopy in Positive HRFQ Participants

**Figure 5:**
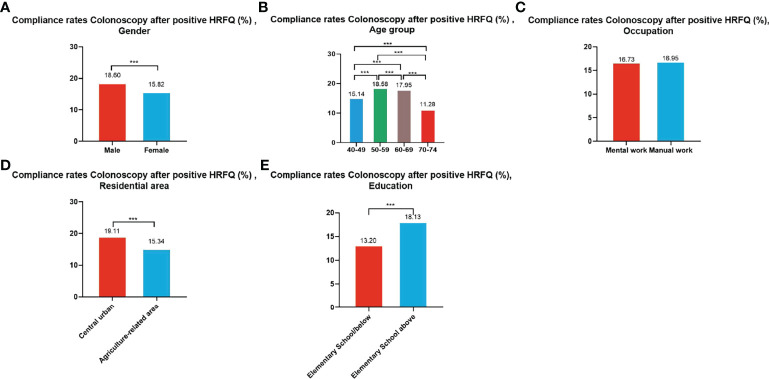
Compliance results by gender **(A)**, age group **(B)**, occupation **(C)**, residential area **(D)**, education **(E)** of subsequent colonoscopy in positive HRFQ participants. ***There are significant differences in detection rates between the different screening methods.

A total of 195,622 participants had positive HRFQ results from 2012 to 2020, of whom 33,177 accepted to undergo colonoscopies; demonstrating a compliance rate of 16.96%. The compliance rate in males (18.60%) was higher than in females (15.82%) (**Table 7** and **Figure 5A**). In descending order, the compliance rate in the 50-59, 60-69, 40-49, and 70-74 age groups was 18.58%, 17.95%, 15.14%, and 11.28%, respectively (**Table 7** and **Figure 5B**). Further, the compliance rate in participants who had primary school bove education level (18.13%) was higher than those with lower education (13.20%) (**Table 7** and **Figure 5E**), and higher in central urban areas (19.11%) than in agriculture-related areas (15.34%) (**Table 7** and **Figure 5D**) but was similar in manual workers (16.95%) and those doing mental work (16.73%) (**Table 7** and **Figure 5C**).

**Table 7:** Analysis of compliance results of subsequent colonoscopy in positive HRFQ participants, (n,%).

Year	Positive HRFQ	Colonoscopy	①^*^ (%)	Gender		Age group				Occupation		Residential area		Education	
				Male	Female	40-49	50-59	60-69	70-74	mental work	manual work	②^*^	③^*^	④^*^	⑤^*^
2012-2014	95016	22290	23.46	9593 (24.96)	12697 (22.44)	2136 (18.99)	6849 (26.01)	11084 (24.84)	2161 (16.84)	7388 (22.49)	14745 (23.76)	10200 (24.69)	12030 (22.40)	4995 (18.74)	17133 (25.11)
2015-2017	49193	7023	14.28	3465 (16.69)	3558 (12.51)	983^#^ (14.41)	2339 (16.42)	3216^#^ (14.08)	485 (9.18)	1671 (12.39)	5349 (14.98)	3396 (17.21)	3627 (12.31)	1031 (8.60)	5989 (16.10)
2018-2020	51413	3864	7.52%	1864 (8.87)	2000 (6.58)	368^#^ (7.40)	1364^#^ (8.40)	1898^#^ (8.32)	234^#^ (3.16)	1175 (7.92)	2613 (7.22)	2191 (10.15)	1673 (5.61)	347 (3.60)	3391 (8.31)
2012-2020	195622	33177	16.96	14922 (18.60)	18255 (15.82)	3487 (15.14)	10552 (18.58)	16198 (17.95)	2880 (11.28)	10234^#^ (16.73)	22707^#^ (16.95)	15787 (19.11)	17330 (15.34)	6373 (13.20)	26513 (18.13)

missing values for occupation are 176; missing values for education are 231;

①^*^: Compliance results of subsequent colonoscopy in positive HRFQ participants; ②^*^:central urban; ③^*^:agriculture-related areas; ④^*^:Elementary School/below; ⑤^*^:Elementary school above;

^#^No significant differences were observed between groups; And statistically significant differences were detected between the other groups.

#### 3.3.4 Compliance Results of Subsequent Colonoscopy in Positive FIT Participants

**Figure 6:**
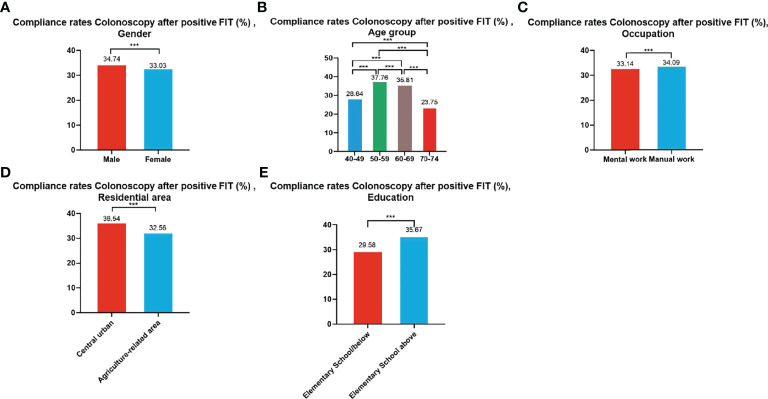
Compliance results by gender **(A)**, age group **(B)**, occupation **(C)**, residential area **(D)**, education **(E)** of subsequent colonoscopy in positive FIT participants. ***There are significant differences in detection rates between the different screening methods.

A total of 99,703 participants had positive FIT results from 2012 to 2020 of whom 33,741 accepted to undergo colonoscopies; demonstrating a compliance rate of 33.84%. The compliance rate of males (34.74%) was higher than in females (33.03%) (**Table 8** and **Figure 6A**). In descending order, the compliance rate of the 50-59, 60-69, 40-49, and 70-74 age groups was 37.76%, 35.81%, 28.64%, and 23.75%, respectively (**Table 8** and **Figure 6B**). Further, the compliance rate in participants who had primary school above education level (35.67%) was higher than those with lower education (29.58%) (**Table 8** and **Figure 6E**), higher in manual workers (34.09%) than in those doing mental work (33.14%) (**Table 8** and **Figure 6C**), and higher in central urban areas (36.54%) than in agriculture-related areas (32.56%) (**Table 8** and **Figure 6D**).

**Table 8:** Analysis of compliance results of subsequent colonoscopy in positive FIT participants, (n,%).

Year	Positive FIT	Colonoscopy	①* (%)	Gender		Age group				Occupation		Residential area		Education	
				Male	Female	40-49	50-59	60-69	70-74	mental work	manual work	②*	③*	④^*^	⑤^*^
2012-2014	37415	13078	34.95	6476 (36.34)	6602 (33.69)	1791 (31.48)	4391 (35.36)	5716 (37.64)	1180 (28.61)	3867 (32.72)	9165 (35.88)	3489 (35.79)	9588 (34.66)	4216 (31.10)	8814 (37.02)
2015-2017	29273	9610	32.83	4730 (33.45)	4880 (32.25)	1676# (31.83)	3133 (39.45)	4107# (31.46)	694 (23.03)	2393# (32.12)	7210# (33.05)	3371# (32.61)	6239# (32.95)	2404 (28.98)	7199 (34.33)
2018-2020	33015	11053	33.48	5262 (34.07)	5791 (32.96)	463 (16.73)	3502 (39.62)	5908 (37.66)	1180 (20.63)	3024 (34.59)	7993 (33.13)	4824 (40.58)	6226 (29.48)	2265 (27.69)	8678 (35.50)
2012-2020	99703	33741	33.84	16468 (34.74)	17273 (33.03)	3930 (28.64)	11026 (37.76)	15731 (35.81)	3054 (23.75)	9284 (33.14)	24368 (34.09)	11684 (36.54)	22053 (32.56)	8885 (29.58)	24691 (35.67)

missing values for occupation are 89; missing values for residential area are 4; missing values for education are 165;

①^*^: compliance results of subsequent colonoscopy in positive FIT participants; ②^*^:central urban; ③^*^:agriculture-related areas; ④^*^:Elementary School/below; ⑤^*^:Elementary school above;

^#^No significant differences were observed between groups; And statistically significant differences were detected between the other groups.

### 3.4 Comparison of Colonoscopy Results Among Different Screening Methods in Tianjin CRC Screening Program

In regards to abnormal lesions identified by colonoscopy, in descending order, the proportion of intestinal diseases detected in the positive FIT group was 33.84%, 22.23% in the high-risk group, 16.96% in the positive HRFQ group, 1.40% overall, and 0.37% in the non-high risk group (**Table 9–1**). Further, the incidence of CRC, AA, and other intestinal diseases was significantly different among the three groups; highest in the positive FIT group, followed by the high-risk group, and lowest in the positive HRFQ group. No significant difference in polyp detection rate was found among the three groups (**Table 9–2** and **Figure 7–1**).

**Figure 7-1:**
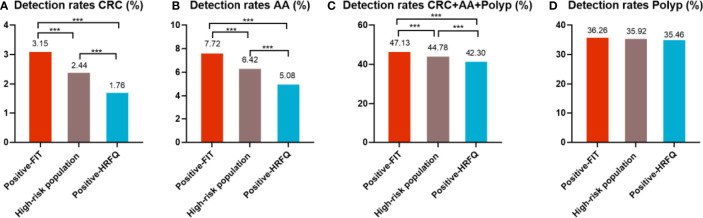
Comparison of colonoscopy results of CRC **(A)**, AA **(B)**, CRC+AA+polyp **(C)** and polyp **(D)** among different screening methods in Tianjin CRC screening program. ***There are significant differences in detection rates between the different screening methods.

**Table 9-1:** Distribution of colonoscopy results among different screening methods in Tianjin CRC screening program (2012-2020), (n,%).

Screening methods	n	Colonoscopy	Colonoscopy (%)	CRC		AA		Polyp		CRC+AA+Polyp	
				n	Detection rate (%)	n	Detection rate (%)	n	Detection rate (%)	n	Detection rate (%)
Positive HRFQ	195622	33177	16.96	585	1.76	1685	5.08	11765	35.46	14035	42.30
Positive FIT	99703	33741	33.84	1062	3.15	2606	7.72	12235	36.26	15903	47.13
High-risk population	279748	62180	22.23	1515	2.44	3994	6.42	22340	35.93	27849	44.79
Non high-risk population	5668238	21059	0.37	478	2.27	1218	5.78	7864	37.34	9560	45.40
Overall	5947986	83239	1.40	1993	2.39	5212	6.26	30204	36.29	37409	44.94

**Table 9-2:** Comparison of colonoscopy results among different screening methods in Tianjin CRC screening program (2012-2020), (n,%).

Screening methods	Colonoscopy (%)	CRC		χ^2^	*P* value	AA		χ^2^	*P* value	Polyp		χ^2^	*P* value	CRC+AA+Polyp		χ^2^	*P* value
		n	Dtection rate (%)			n	Dtection rate (%)			n	Dtection rate (%)			n	Dtection rate (%)		
Positive HRFQ	16.96	585	1.76			1685	5.08			11765	35.46			14035	42.30		
Positive FIT	33.84	1062	3.15	134.243	<0.001	2606	7.72	194.839	<0.001	12235	36.26	4.705	0.095	15903	47.13	157.807	<0.001
High-risk population	22.23	1515	2.44			3994	6.42			22340	35.93			27849	44.79		

Positive FIT detected the highest CRC regardless of HRFQ results; while for AA, intestinal disease, positive FIT only was the highest detection rate, followed by positive HRFQ & FIT; for polyps, no difference was seen between the detection rates regardless of HRFQ and FIT results (**Tables 9–3**, **9–4** and **Figure 7–2**). Combined with our screening strategy, where the high-risk group is either positive HRFQ or positive FIT, the different combinations of HRFQ and FIT mentioned above are included in the screening, which can enroll more high-risk patients than FIT or HRFQ only, and thus detect more intestinal diseases (**Table 9–5** and **Figure 7–3**). In a separate study, the sensitivity of predicting CRC after colonoscopy in the high-risk group was 76.02%; the specificity was 25.33% (**Table 9–6**).

**Table 9-3:** Distribution of colonoscopy results among different screening methods in Tianjin CRC screening program (2012-2020), (n, %).

Screening methods	n	Colonoscopy	Colonoscopy (%)	CRC		AA		Polyp		CRC+AA+Polyp	
				n	Detection rate (%)	n	Detection rate (%)	n	Detection rate (%)	n	Detection rate (%)
HRFQ(-)+N0-FIT	1244028	14559	1.17	365	2.51	778	5.34	5035	34.58	6178	42.43
HRFQ(-)+FIT(-)	4424210	6500	0.15	113	1.74	440	6.77	2829	43.52	3382	52.03
HRFQ(-)+FIT(+)	84126	29063	34.55	930	3.20	2309	7.94	10575	36.39	13814	47.53
HRFQ(+)+N0-FIT	63289	146	0.23	49	33.56	32	21.92	46	31.51	127	86.99
HRFQ(+)+FIT(-)	116756	28293	24.23	404	1.43	1356	4.79	10059	35.55	11819	41.77
HRFQ(+)+FIT(+)	15577	4678	30.03	132	2.82	297	6.35	1660	35.49	2089	44.66

**Table 9-4:** Comparison of colonoscopy results among different screening methods in Tianjin CRC screening program (2012-2020), (n,%).

Screening methods	Colonoscopy (%)	CRC		χ^2^	*P* value	AA		χ^2^	*P* value	Polyp		χ^2^	*P* value	CRC+AA+Polyp		χ^2^	*P* value
		n	Dtection rate (%)			n	Dtection rate (%)			n	Dtection rate (%)			n	Dtection rate (%)		
HRFQ(+)+FIT(+)	30.03	132	2.82##	214.928	<0.001	297	6.35#	238.433	<0.001	1660	35.49*※	150.503	<0.001	2089	44.66	319.703	<0.001
HRFQ(+)+FIT(-)	24.23	404	1.43^#^			1356	4.79			10059	35.55#※			11819	41.77		
HRFQ(-)+FIT(-)	0.15	113	1.74^#^			440	6.77#			2829	43.52			3382	52.03		
HRFQ(-)+FIT(+)	34.55	930	3.20##			2309	7.94			10575	36.39#*			13814	47.53		

^#/##/*/※^ No significant differences were observed between Screening methods.

**Table 9-5:** Comparison of colonoscopy results for different combinations of screening methods for HRFQ and FIT, (n, %).

Screening methods	CRC		AA		Polyp		CRC+AA+Polyp	
	n	Dtection rate (%)	n	Dtection rate (%)	n	Dtection rate (%)	n	Dtection rate (%)
HRFQ(-)+No FIT	365	0.44	778	0.93	5035	6.05	6178	7.42
HRFQ(+)+No FIT	49	0.06	32	0.04	46	0.06	127	0.15
HRFQ(+)+FIT(+)	132	0.16	297	0.36	1660	1.99	2089	2.51
HRFQ(+)+FIT(-)	404	0.49	1356	1.63	10059	12.08	11819	14.20
HRFQ(-)+FIT(-)	113	0.14	440	0.53	2829	3.40	3382	4.06
HRFQ(-)+FIT(+)	930	1.12	2309	2.77	10575	12.70	13814	16.60
HRFQ(+)/FIT(+)	1515	1.82	3994	4.80	22340	26.84	27849	33.46
All colonoscopy	83239							

High-risk population represented by HRFQ(+)/FIT(+), HRFQ(+)/FIT(+)=HRFQ(+)+ FIT(+)、HRFQ(+)+ FIT(-)、HRFQ(-)+ FIT(+)、HRFQ(+)+No-FIT;HRFQ(+)/FIT(+); Dtection rate (%)=Screening methods(such as HRFQ(-)+FIT(+))/All colonoscopy*100%.

**Table 9-6:** Analysis of CRC detected in high-risk groups versus CRC diagnosed by colonoscopy results, n (%).

			Colonoscopy		
			CRC	Non-CRC	
**High-risk group**	**CRC**		1515	60665	62180
	**Non-CRC**		478	20581	21059
			1993	81246	83239

Sensitivity (Predicting CRC sensitivity in high-risk population)=1515/1993*100%=76.02%; Specificity (Predicting CRC Specificity in high-risk population)=20581/81246 = 25.33%.

**Figure 7-2:**
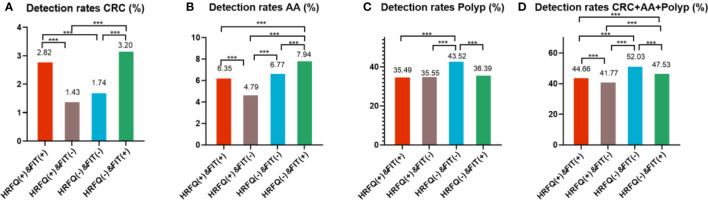
Comparison of colonoscopy results of CRC **(A)**, AA **(B)**, CRC+AA+polyp **(C)** and polyp **(D)** among different screening methods in Tianjin CRC screening program. ***There are significant differences in detection rates between the different screening methods.

**Figure 7-3:**
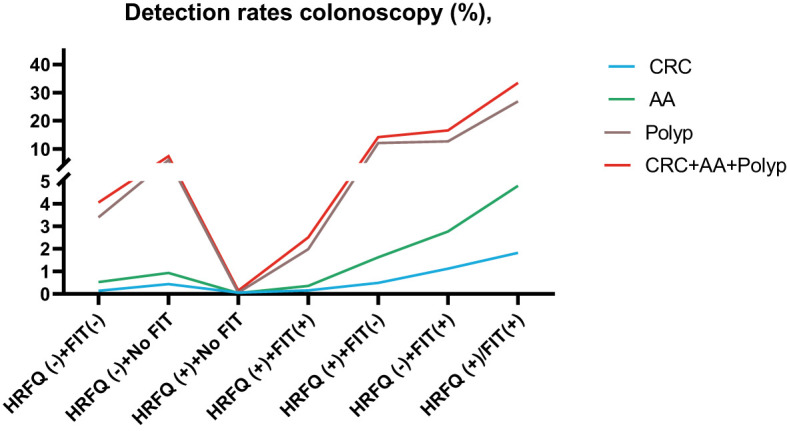
Comparison of colonoscopy results among different screening methods in Tianjin CRC screening program.

In addition, it is worthwhile to pay attention to the fact that the screening results of negative HRFQ & FIT have the highest detection rate of intestinal disease and the second highest detection rate of AA, and this group of patients has poor compliance (voluntary colonoscopy and lack of physician recommendation), which requires additional attention in the follow-up study (**Tables 9–3**, **9–4** and **Figure 7–2**).

### 3.5 Evaluation of the Diagnostic Performance of Colonoscopy

#### 3.5.1 Disease Detection in the Population Undergoing Colonoscopy

**Figure 8:**
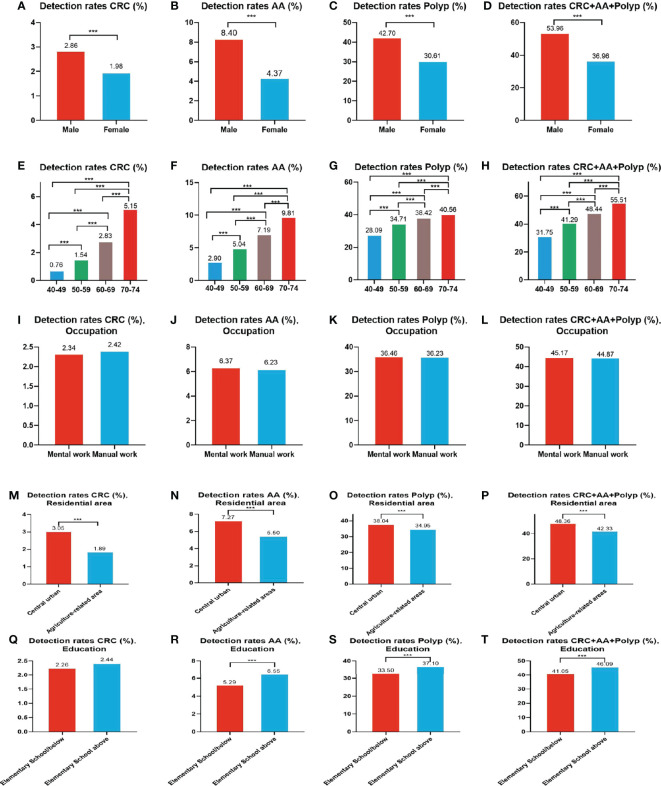
Evaluation of the diagnostic performance of colonoscopy for CRC **(A, E, I, M, Q)**, AA **(B, F, J, N, R)**, CRC+AA+polyp **(C, G, K, O, S)** and polyps **(D, H, L, P, T)**. ***There are significant differences in detection rates between the different screening methods.

A total of 1,993 CRC cases were detected in residents who accepted colonoscopy. CRC detection rate in males (2.86%) was higher than in females (1.98%) (**Table 10** and **Figure 8A**). The detection rate in the 40-49, 50-59, 60-69, and 70-74 age groups was 0.76%, 1.54%, 2.83%, and 5.15% (**Table 10** and **Figure 8E**), respectively, and was also higher in central urban areas (3.05%) than in agriculture-related areas (1.89%) (**Table 10** and **Figure 8M**).

**Table 10:** Evaluation of the diagnostic performance of colonoscopy, (n,%).

	n	Detection rate (%)	Gender		Age group				Occupation		*P* value	Residential area		*P* value	Education		*P* value
			Male	Female	40-49	50-59	60-69	70-74	mental work	manual work		②^*^	③^*^		④^*^	⑤^*^	
CRC	1993	2.39	1119 (2.86)	874 (1.98)	70 (0.76)	408 (1.54)	1134 (2.83)	381 (5.15)	589# (2.34)	1393# (2.42)	0.473	1098 (3.05)	894 (1.89)	0.000	413# (2.26)	1569# (2.44)	0.152
AA	5212	6.26	3281 (8.40)	1931 (4.37)	267 (2.90)	1337 (5.04)	2882 (7.19)	726 (9.81)	1605# (6.37)	3583# (6.23)	0.432	2614 (7.27)	2598 (5.50)	0.000	968 (5.29)	4208 (6.55)	0.000
Polyp	30204	36.29	16683 (42.70)	13521 (30.61)	2588 (28.09)	9214 (34.71)	15399 (38.42)	3003 (40.56)	9187# (36.46)	20849# (36.23)	0.520	13683 (38.04)	16518 (34.95)	0.000	6130 (33.50)	23850 (37.10)	0.000
CRC+AA+Polyp	37409	44.94	21083 (53.96)	16326 (36.96)	2925 (31.75)	10959 (41.29)	19415 (48.44)	4110 (55.51)	11381# (45.17)	25825# (44.87)	0.433	17395 (48.36)	20010 (42.33)	0.000	7511 (41.05)	29627 (46.09)	0.000

missing values for occupation are 406; missing values for residential area are 8; missing values for education are 542;

②^*^:central urban; ③^*^:agriculture-related areas; ④^*^:Elementary School/below; ⑤^*^:Elementary school above;

^#^No significant differences were observed between groups; And statistically significant differences were detected between the other groups.

A total of 5,212 AA cases were detected in residents who accepted colonoscopy. AA detection rate in males (8.40%) was higher than in females (4.37%) (**Table 10** and **Figure 8B**). The detection rate in the 40-49, 50-59, 60-69, and 70-74 age groups was 2.90%, 5.04%, 7.19%, and 9.81%, respectively(**Table 10** and **Figure 8F**). Further, the AA detection rate in central urban areas (7.27%) was higher than in agriculture-related areas (5.50%) (**Table 10** and **Figure 8N**) and higher in those with primary school above education level (6.55%) than those with lower education (5.29%) (**Table 10** and **Figure 8R**).

A total of 30,204 polyps cases were detected in residents who accepted colonoscopy. The polyps detection rate in males (42.70%) was higher than in females (30.61%) (**Table 10** and **Figure 8C**), and was 28.09%, 34.71%, 38.42%, and 40.56% for the 40-49, 50-59, 60-69, and 70-74 age groups, respectively (**Table 10** and **Figure 8G**). Further, the detection rate was higher in central urban areas (38.04%) than in agriculture-related areas (34.95%) (**Table 10** and **Figure 8O**), and higher in those with primary school above education level (37.10%) than those with lower education level (33.50%) (**Table 10** and **Figure 8S**).

A total of 37,409 intestinal diseases were detected in residents who accepted colonoscopy. The intestinal diseases detection rate in males (53.96%) was higher than in females (36.96%) (**Table 10** and **Figure 8D**) and was 31.75%, 41.29%, 48.44%, and 55.51% for the 40-49, 50-59, 60-69, and 70-74 age groups (**Table 10** and **Figure 8H**), respectively. Further, the detection rate was higher in central urban areas (48.36%) than in agriculture-related areas (42.33%) (**Table 10** and **Figure 8P**), and higher in those with primary school above education level(46.09%) than those with lower education level (41.05%) (**Table 10** and **Figure 8T**).

In addition, the detection rate of CRC, AA, polyps, and intestinal disease was no higher in manual workers than in those engaged in mental work (**Table 10** and **Figures 8I-L**). CRC detection rates were slightly higher among those with primary school above education level than those with lower education, but no significant differences were seen (**Table 10** and **Figure 8Q**).

#### 3.5.2 Staging and Location Distribution Characteristics of Different Screening Methods

Of the 1993 CRC cases, 1571 had no T-stage while 422 had clear T-stage, accounting for 21.17%. stage II (40.52%) was the most common CRC stage, and since information on CRC stage and site was less available, only a proportional analysis was performed in this study, and The staging and location distribution characteristics of the different screening methods are described below and can be seen in **Table 11**.

**Table 11:** Staging and location distribution characteristics of different screening methods, (n).

Screening methods	CRC		I-II		III-IV		left colon/rectum		right colon	
	n	Dtection rate	n	Dtection rate	n	Dtection rate	n	Dtection rate	n	Dtection rate
HRFQ(-)+No FIT	365	2.51%	62	0.43%	45	0.31%	81	0.56%	25	0.17%
HRFQ(+)+No FIT	49	33.56%	13	8.90%	8	5.48%	11	7.53%	10	6.85%
HRFQ(+)+FIT(+)	132	2.82%	12	0.26%	8	0.17%	16	0.34%	4	0.09%
HRFQ(+)+FIT(-)	404	1.43%	28	0.10%	11	0.04%	26	0.09%	13	0.05%
HRFQ(-)+FIT(-)	113	1.74%	14	0.22%	5	0.08%	14	0.22%	5	0.08%
HRFQ(-)+FIT(+)	930	3.20%	136	0.47%	80	0.28%	153	0.53%	62	0.21%
HRFQ(+)/FIT(+)	1515	2.44%	189	0.30%	107	0.17%	206	0.33%	89	0.14%

#### 3.5.3 Analysis of CRC/AA Detection Rate in Three Cycles of Screening

**Figure 9:**
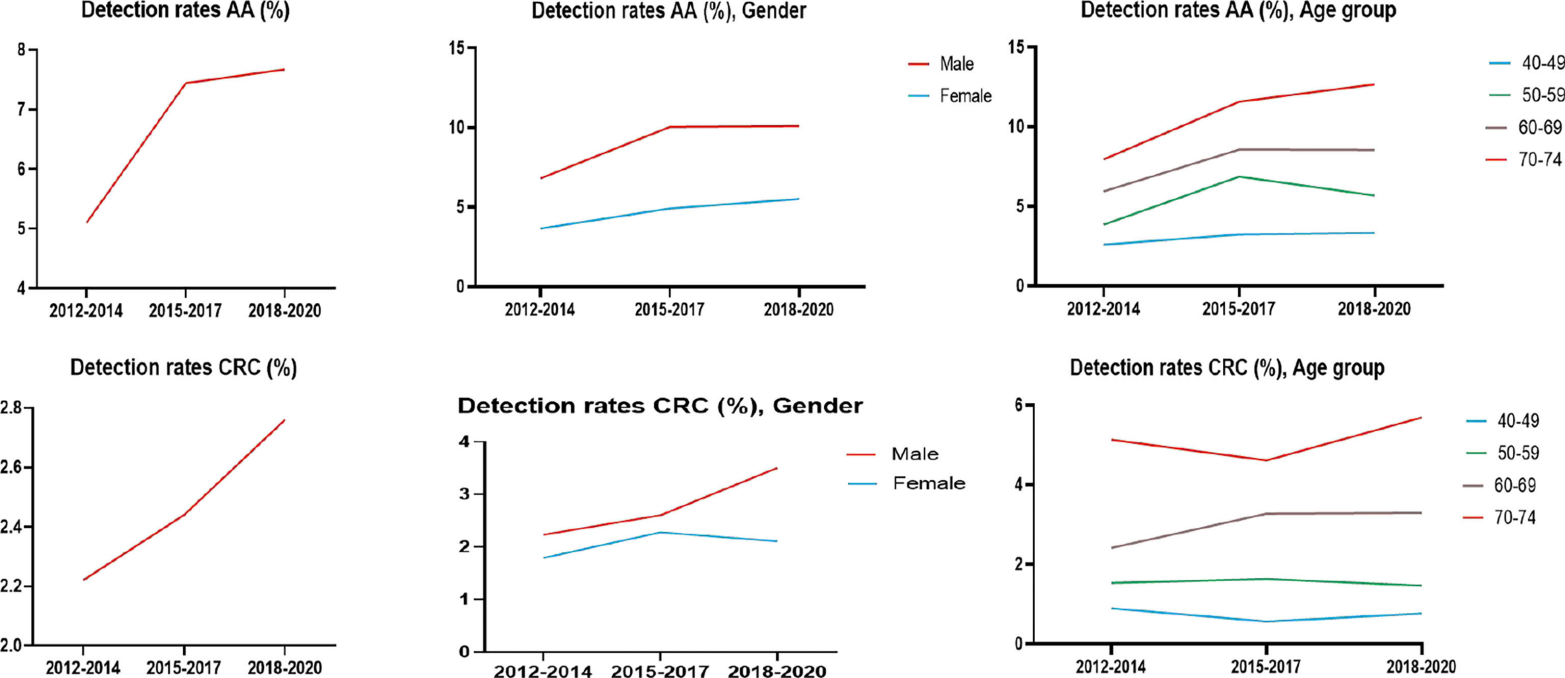
Analysis of CRC/AA detection rate in 3 cycles of screening.

The detection rates of CRC and AA in cycles 2 and 3 were higher than those in cycle 1. Further analysis revealed no significant increase in the detection rate of CRC in people under 40-49 years of age during the 3 cycles of screening, and the detection rate of their AA showed an increasing trend, but there was no significant differences (**Tables 12–1**, **12–2**, and **Figure 9**).

**Table 12-1:** Analysis of CRC detection rate in 3 cycles of screening, (%).

		2012-2014	2015-2017	2018-2020
CRC		2.22#	2.44#*	2.76*
Gender				
	Male	2.23#	2.60#	3.50
	Female	1.79#	2.28#	2.11#
Age group				
	40-49	0.89#	0.56#	0.76#
	50-59	1.53#	1.63#	1.46#
	60-69	2.41	3.27#	3.29#
	70-74	5.13#	4.61#	5.69#
Gender Distribution in Different Age Groups				
40-49	Male	0.89#	0.62#	0.90#
	Female	0.89#	0.49#	0.61#
50-59	Male	2.09#	1.67#	1.74#
	Female	1.11#	1.60#	1.25#
60-69	Male	2.95*	3.53#*	4.16#
	Female	1.94*	3.01#	2.48#*
70-74	Male	5.31#	4.35#	6.75#
	Female	4.92#	4.92#	4.49#

^#/*^ No significant differences were observed between Screening methods; And statistically significant differences were detected between the other groups.

**Table 12-2:** Analysis of AA detection rate in 3 cycles of screening, (%).

		2012-2014	2015-2017	2018-2020
AA		5.10	7.44#	7.67#
Gender				
	Male	6.81	10.04#	10.09#
	Female	3.65	4.92#	5.52#
Age group				
	40-49	2.59#	3.23#	3.33#
	50-59	3.85	6.87	5.68
	60-69	5.94	8.57#	8.54#
	70-74	7.95	11.58#	12.67#
Gender Distribution in Different Age Groups				
40-49	Male	3.71#	4.62#	4.48#
	Female	1.66#	1.85#	2.03#
50-59	Male	5.59	9.82	7.76
	Female	2.57	4.35#	4.15#
60-69	Male	7.74	11.30#	11.27#
	Female	4.35	5.81#	5.98#
70-74	Male	9.02	13.87#	14.27#
	Female	6.77#	8.85#*	10.86*

^#/*^ No significant differences were observed between Screening methods; And statistically significant differences were detected between the other groups.

## 4 Discussion

The findings of this study are based on a mass screening performed in community hospitals in the Tianjin city of China which screened nearly six million asymptomatic individuals from 2012 to 2020; representing the largest CRC screening dataset analyzed in current literature. These data could be used as a reference to countries that plan to conduct population-based CRC screening.

The effectiveness of screening not only depends on its characteristics but also on the compliance of the participants. The screening protocol used in this study was proposed by the China Health Commission, which combined the use of HRFQ and FIT, followed by colonoscopy when necessary. Although all participants (N = 5,947,986) in the first screening stage completed the HRFQ, only 78% underwent FIT, but still this FIT completion rate was higher than those of other national screening programs which used FOBT and FIT (range, 42%-70%) (19, 20). This could be because, in this screening process, the investigators first used the HRFQ which could effectively identify high-risk individuals who were then given professional guidance by physicians to undergo FIT. This rationale is consistent with that of Chen et al. who reported that multiple interventions could have a more positive impact on the compliance of participants to undergo colonoscopy and FOBT, compared with single intervention screening (21).

Further, on the one hand, we found that women had both higher HRFQ completion rate and FIT compliance rate than men, indicating that women could be more likely to accept the physicians’ or investigators’ initial recommendations (i.e., more obedient to authorities and experts) but on the other hand, we also observed that the positive rate of FIT and compliance rate for subsequent colonoscopy were significantly higher in males than in females; demonstrating a gender difference in the preference of screening tools, which was consistent with that observed in a study from Korea (22). Thus, it could be deduced that compared with the relatively simple FOBT/FIT, females might feel more reluctant to undergo colonoscopy due to traditional beliefs and their own physiological characteristics or discomforts. Besides, the detection rate of intestinal diseases from colonoscopy was higher in men than in women, which could be related to the higher CRC incidence in males (23). Therefore, researchers should make more efforts to increase awareness for CRC screening in men and should also find more re-assuring ways to motivate more women to undergo colonoscopy, which could therefore improve the overall compliance rate of colonoscopy and screening effectivity.

The incidence of CRC has been also reported to be on the rise in people under 50 years of age in many high-income countries such as Australia, Canada, Germany and the United Kingdom (24). There have been opportunistic CRC screenings in people aged 40 years and older in Austria since 1980s. In 2018, the American Cancer Society proposed new CRC screening recommendations which suggested lowering the starting age for CRC screening, from 50 to 45 years old, based on the estimated average risk for CRC. However, some studies have suggested even lower screening starting age, i.e., 40 years old (25, 26). Such opinion is also supported by colorectal cancer statistics from the United States, which were performed in 2020 and showed an increased incidence of CRC in younger people. We further analyzed the participants aged 40-49 years in this study and found that no increased detection of CRC was observed in this group of younger people, but found that the detection of AA showed an increasing trend, and thus it is possible that CRC has an increased risk in the development of young people. Further, we also found that HRFQ positive rate, FIT positive rate and intestinal diseases detection rate were associated with aging. People aged 50-59 years old had the highest compliance rate for FIT after HRFQ and also for subsequent colonoscopy in the high-risk population, while the 70-74 age group had the lowest compliance rate. Considering that the risk of colonoscopy-related complications might be higher in elderly people, this low compliance rate could therefore be attributed to a fear of colonoscopy in older adults, and thus, colonoscopy under anesthesia could be recommended for such individuals. Meanwhile, the U.S. Preventive Services Task Force also suggested that CRC screening in adults over 75 years of age should vary from person to person, depending on their personal health status and previous screening history (27). Therefore, it is crucial to balance the risk-to-benefit ratio of colonoscopy in older people in screening programs.

Further, we observed that participants with primary school above education level, resided in central urban areas, and who were manual workers were more compliant to the screening program and had higher subsequent colonoscopy compliance rate. As such, we observed that the detection rate of intestinal diseases was higher among those with primary school above education level and were from central urban areas; suggesting a possible association between higher education level, awareness of cancer prevention, self-care and adherence to researchers’ guidance. A previous study indicated potential associations between low education or income level and knowledge or awareness of cancer prevention (28). In this regard, due to the relatively poorer understanding of cancer prevention and lesser literacy rate or income of rural residents in China, this could make them more susceptible to undiagnosed or late-treatment of intestinal diseases (29). Besides, in recent years, improving the efficacy of CRC screening in agriculture-related areas were given great importance because of growing disease incidence and mortality. Thus, organized cancer screening programs funded by the Chinese government have been implemented in rural and urban areas of China, aiming to solve the accessibility of colonoscopy dilemma in agriculture-related areas through a series of publicity plans on cancer prevention awareness, the inclusion of colonoscopy in medical insurance policies and offering of free colonoscopy. Such initiatives are providing more concrete evidence of the real situation of intestinal diseases in agriculture-related areas and people with lower education level, and are also helping those in need. Thus, authorities should continue to increase these efforts for less educated people in subsequent vulnerable regions. Further, it was reported that the incidence of CRC in China was positively correlated with China’s gross domestic product per capita (GDPPC) level (30), which reasonably explain the higher incidence of intestinal diseases among mental workers as they usually have better economic level, live a more modern lifestyle, and perform less physical activities.

The main weakness of the screening program was the low compliance rate for colonoscopy in participants who were positive after the first screening stage. This might have affected the effectiveness of the CRC screening by lowering the actual CRC or adenoma detection rate. Thus, another aim of this study was to assess the examination rate and diagnostic utility of colonoscopy in different screening methods in the Tianjin CRC screening program. We observed that the compliance rate of colonoscopy among the high-risk group in our study was 22.23%, which was higher than that observed in other studies (range, 14.0%-18.7%) (21, 31, 32). Of these, the compliance rate for colonoscopy among positive FIT participants was 33.84%; lower than that observed in a prior study (78.4%) (33), while the compliance rate for colonoscopy among positive HRFQ participants was 16.96%. Further analyses showed that the detection rate of intestinal diseases among the high-risk group, FIT positive group and HRFQ positive group were more than 42.0%; higher than that observed in other related studies (33, 34).

At present, FIT is still recognized as one of the most convenient and effective CRC screening methods based on the close relationship between positive FIT and mortality due to multiple causes other than CRC (35, 36). In this study, the detection rates of CRC and AA among the positive FIT group was significantly lower than those in previous reports, while the detection rate of polyps was significantly higher (37). Further, we also observed no difference in polyps detection rate was among participants in the HRFQ positive group, FIT positive group and high-risk group. Moreover, since we observed that the number of participants completing HRFQ was much greater than FIT and the proportion of participants who underwent colonoscopy was higher in those who had positive HRFQ results, compared to those with positive FIT results, these suggest that similar questionnaires could be implemented as a supplementary method for screening polyps to improve screening compliance rate and increasing polyps detection rate.Screening strategies based on HRFQ and FIT can effectively monitor lesions such as CRC and AA, but still suffer from problems such as low positive predictive values, which inevitably increase the workload of colonoscopy. In contrast, some new screening tools, such as monitoring markers in faeces such as SDC2 methylation levels (38), miRNA (39) and bacteria in faeces (40), can improve the positive predictive value of screening and hold promise as a means of widespread early screening for CRC.

In contrast to other relevant studies which also investigated the detection rate of intestinal diseases in a high-risk population, the detection rate of CRC in this present study was 2.44%, which was higher than that in Shanghai (2.3%) (34), Jiashan (1.2%) (41), Guangzhou (1.17%) (42), but lower than another study in Guangzhou (3.3%) (32). The detection rate of advanced adenomas in this study was 6.42%; higher than that reported in Jiashan (4.4%) but lower than reported in Shanghai (9.3%) and Guangzhou (9.2-9.8%) (32). Further, the detection rate of polyps in this current study was 35.92%, which was higher than in Jiashan (10.7%) and Guangzhou (21.1%-36.3%) (32, 42). Thus, the CRC screening performed in nearly six million asymptomatic people in Tianjin was not only advantageous in detecting CRC but may have also contributed greatly to CRC prevention.

When interpreting our data, specific strengths and limitations should be considered. A major advantage is that our data come from a large population-based colorectal cancer screening program in China. In addition, strict standards are applied to ensure the quality of the research data. However, this study has some limitations. First, our data are derived from a single region, and selection bias cannot be ruled out. Second, despite the large sample size, colonoscopy compliance is low and there may be bias. Third, clinical information on CRC patients is not yet fully available. We only conducted a preliminary analysis of the existing data, and we will try to improve the relevant information as much as possible in the future, and conduct a more in-depth and detailed analysis of the specific problems of screening, so as to provide a basis for the optimization of the screening strategy.

Studies have shown that regardless of the screening strategy chosen, participation compliance remains a key determinant of a screening program’s success (43). In this study, nearly six million participants were screened, of whom all completed the HRFQ and 78% complied to undergo FIT; demonstrating a high compliance rate and representing one of the largest datasets of CRC screening program. Thus, the reported findings could be of certain representative significance. However, one of the limitations observed was that although HRFQ could be more convenient and accepted than FIT, and was associated with a higher detection rate of polyps, it could be less efficient in detecting CRC and advanced adenomas. Further, considering that 0.37% of the participants in the non-high-risk group underwent colonoscopy and had similar detection rates of each intestinal disease as those in the FIT positive group, HRFQ positive group and high-risk group, therefore, the contents of HRFQ should be further optimized based on this study’s findings in order to maximize the value of HRFQ in CRC screening.

## Data Availability Statement

The original contributions presented in the study are included in the article. Further inquiries can be directed to the corresponding author.

## Ethics Statement

The studies involving human participants were reviewed and approved by the local ethical committee in the Health Bureau of Tianjin City. Written informed consent for participation was not required for this study in accordance with the national legislation and the institutional requirements.

## Author Contributions

Conceptualization, XZ, SZha, MZ, and LZ; methodology, MZ, LZ, YDZ, LW, and ZL; software, HZ and YZ; validation, MZ and HJ; formal analysis, MZ, LZ, and YDZ; investigation, LZ, MZ, YDZ, and HJ; resources, SZhu, XZ, and MZ; data curation, HZ, YZ, YDZ, LW, and ZL; writing—original draft preparation, MZ and YDZ; writing—review and editing, SZha, MZ, and LZ; visualization, SZhu and SZha; supervision, XZ and SZha; project administration, MZ and LZ; funding acquisition, SZhu, XZ, and MZ. All authors have read and agreed to the published version of the manuscript.

## Funding

This study was funded by Foundation of Tianjin Union Medical Center (grant number: 2016YJZD002 and 2016RMNK002). This work was funded by Tianjin Key Medical Discipline (Specialty) Construction Project.

## Conflict of Interest

The authors declare that the research was conducted in the absence of any commercial or financial relationships that could be construed as a potential conflict of interest.

## Publisher’s Note

All claims expressed in this article are solely those of the authors and do not necessarily represent those of their affiliated organizations, or those of the publisher, the editors and the reviewers. Any product that may be evaluated in this article, or claim that may be made by its manufacturer, is not guaranteed or endorsed by the publisher.
